# Real-life evidence of encorafenib plus binimetinib in patients with unresectable advanced or metastatic BRAF^V600^-mutant melanoma in Spain: the BECARE (GEM-2002) trial

**DOI:** 10.3389/fonc.2025.1466185

**Published:** 2025-02-26

**Authors:** Ainara Soria, Pedro Sanchez Mauriño, Juan José Serrano Domingo, Regina García Galindo, Silvia Sequero, Lourdes Gutiérrez Sanz, Guillermo Crespo, Roberto Díaz-Beveridge, Teresa Puértolas, Pedro López, Joaquín Fra Rodríguez, Rafael López Castro, Cristina Aguayo, Javier Valdivia, Alberto Jacobo Cunquero-Tomás, Gretel Benítez, Pablo Ayala de Miguel, Enrique Espinosa, Eva Muñoz-Couselo, Begoña Campos, Lourdes García Sánchez, Pablo Cerezuela-Fuentes

**Affiliations:** ^1^ Medical Oncology, Hospital Universitario Ramón y Cajal, Madrid, Spain; ^2^ Medical Oncology, Hospital Universitario Reina Sofia, Córdoba, Spain; ^3^ Medical Oncology, Hospital Universitario de Jerez de la Frontera, Jerez de la Frontera, Spain; ^4^ Medical Oncology, Hospital Universitario San Cecilio, Granada, Spain; ^5^ Medical Oncology, Hospital Universitario Puerta de Hierro, Majadahonda, Spain; ^6^ Medical Oncology, Hospital Universitario de Burgos, Burgos, Spain; ^7^ Medical Oncology, Hospital Universitario y Politécnico la Fe de Valencia, Valencia, Spain; ^8^ Medical Oncology, Hospital Universitario Miguel Servet, Zaragoza, Spain; ^9^ Medical Oncology, Complejo Hospitalario de Jaén, Jaén, Spain; ^10^ Medical Oncology, Hospital Universitario Río Hortega, Valladolid, Spain; ^11^ Medical Oncology, Hospital Clínico Universitario de Valladolid, Valladolid, Spain; ^12^ Medical Oncology, Hospital Universitario Infanta Sofía, San Sebastián de los Reyes, Spain; ^13^ Medical Oncology, Hospital Universitario Virgen de las Nieves, Granada, Spain; ^14^ Medical Oncology, Consorcio Hospital General Universitario de Valencia, Valencia, Spain; ^15^ Medical Oncology, Complejo Hospitalario Universitario Insular-Materno infantil de Gran Canaria, Las Palmas de Gran Canaria, Spain; ^16^ Medical Oncology, Hospital Universitario San Pedro de Alcántara, Cáceres, Cáceres, Spain; ^17^ Universidad Autónoma de Madrid, School of Medicine - Hospital Universitario La Paz - CIBERONC - Madrid, Madrid, Spain; ^18^ Medical Oncology, Hospital Universitario Vall d’Hebron & Vall d’Hebron Institute of Oncology (VHIO), Barcelona, Spain; ^19^ Medical Oncology, Hospital Universitario Lucus Augusti de Lugo, Lugo, Spain; ^20^ Medical Oncology, Complejo Asistencial de Segovia, Segovia, Spain; ^21^ Medical Oncology, Hospital Clínico Universitario Virgen de la Arrixaca, Instituto Murciano de Investigación Biosanitaria (IMIB)-Arrixaca, Ciudad de Murcia, Spain

**Keywords:** melanoma, molecular targeted therapy, mutation, retrospective studies, immune checkpoint inhibitors

## Abstract

**Purpose:**

Combined BRAF/MEK inhibition with encorafenib (E) plus binimetinib (B) has demonstrated efficacy and tolerability in phase III clinical trials, and is the standard of care for advanced/metastatic BRAF^V600^-mutant melanoma. However, real-life evidence is limited, particularly in patients pre-treated with immune checkpoint inhibitors (ICI).

**Patients and methods:**

BECARE GEM 2002 was a retrospective, non-interventional study aimed at investigating the real-world effectiveness and tolerability of EB in patients with unresectable or metastatic BRAF^V600^-mutant melanoma conducted at 21 sites in Spain. The primary objective of this study was to characterise the population of patients receiving EB and assess the efficacy and tolerability of EB in real life. The study included patients treated according to standard clinical practice with EB as the 1^st^ line or 2^nd^ line after progression to ICI for an unresectable or metastatic stage. Patients who previously received treatment with BRAF and/or MEK inhibitor, other than as adjuvants, that ended ≥ 6 m before EB were not eligible

**Results:**

From September 2021 to March 2023, 117 patients were included; 89 (76.1%) and 28 (23.9%) patients received EB as 1^st^ line and 2^nd^ line, respectively. The median follow-up was 13.8 months (95% CI: 12.0-17.4). In patients with EB as 1^st^ line treatment, ORR and median PFS were 75% and 12 months (95% CI: 9.4-18.6), respectively. In patients with EB as 2^nd^ line treatment after ICI, ORR and median PFS were 77.8% and 12.5 months (95% CI: 6.6-NA), respectively. In patients with brain metastasis ORR and median PFS were 70.8% and 6.3 months (95% CI: 6.1-10.3). Treatment-related adverse events of grade ≥3 were reported in 17 (14.5%) patients; transaminitis (9.4%) and diarrhoea (2.6%) were the most frequent adverse events.

**Conclusion:**

In this real-world study, EB treatment demonstrated effectiveness and a consistent safety profile in patients with BRAF^V600^-mutant melanoma treated according to standard clinical practice, including in those with prior ICI treatment and of brain metastasis; therefore, EB is a feasible treatment option for unresectable and metastatic melanoma.

Clinical trial identification: REec: 0004-2021-OBS

**Clinical trial identification:**

REec: 0004-2021-OBS.

## 1 Introduction

In the last decade, BRAF- and MEK-targeted therapies (TT) and anti-CTLA4 and anti-PD1 immune checkpoint inhibitor antibodies (ICIs) (1) have been introduced to improve the prognosis of patients with advanced and metastatic melanoma. These therapies represent the current standard of care for patients with locally advanced, unresectable, or metastatic BRAF^V600^-mutant melanoma.

Anti-PD1 pembrolizumab therapy achieved a 5-year overall survival (OS) rate of 38.7% (2) and combined anti-PD1 and anti-CTLA4 treatment with nivolumab and ipilimumab resulted in 5-year OS and progression-free survival (PFS) rates of 44 and 29%, respectively (3). Additionally, three TT combinations of BRAF and MEK inhibitors are available: encorafenib plus binimetinib, dabrafenib plus trametinib and vemurafenib plus cobimetinib. Comparisons between these clinical trials are limited, and only vemurafenib monotherapy has been used as a comparator in different phase III studies (4–6), showing that the combination of encorafenib plus binimetinib has a comparable or improved efficacy. The 5-year OS rates were 35%, 34% and 31%, and the observed median OS was 33.6, 25.9 and 22.5 months for encorafenib plus binimetinib (7), dabrafenib plus trametinib (8) and vemurafenib plus cobimetinib (9), respectively. The safety profiles of the three combinations were similar, with a combination-specific increase in punctual and generally mild adverse events such as fever for dabrafenib plus trametinib; skin toxicity, vemurafenib plus cobimetinib; neuromonopathies, encorafenib plus binimetinib.

After marketing authorisation by the European Medicines Agency (EMA) for the treatment of adult patients with unresectable or metastatic melanoma with the BRAF^V600^ mutation, encorafenib plus binimetinib became the standard of care for patients, including those with diverse profiles and prognostic characteristics who were excluded from the phase III registration trial. For instance, less than 5% of patients in the COLUMBUS trial had received check-point inhibitors prior to study inclusion, and only nine patients (4.7%) had brain metastasis.

Real-life evidence of the efficacy and tolerability of encorafenib plus binimetinib is limited, particularly in patients pre-treated with immune checkpoint inhibitors.

This study investigated the real-world efficacy, safety and tolerability of encorafenib plus binimetinib in unresectable advanced or metastatic BRAF^V600^-mutant malignant melanoma in Spain, focusing on patients who were treated in the 1^st^ line and in the 2^nd^ line after receiving ICIs.

## 2 Materials and methods

### 2.1 Study design

BECARE is a multicentre, retrospective, non-interventional, observational study of encorafenib plus binimetinib in patients diagnosed with unresectable or metastatic (TNM Classification, 8^th^ edition) BRAF^V600^-mutant melanoma conducted at 21 sites in Spain. All data documented in the study were obtained from the patients’ medical history and the decision to treat the patients with a specific therapeutic strategy was made before and independently from inclusion in the study, following standard clinical practice. Data from patients’ clinical histories were collected retrospectively for at least 12 months. Survival follow-up was extended until at least 51% of the documented patients declared exitus to ensure sufficient data maturity for time-to-event endpoints. All patients with melanoma who met the eligibility criteria at the participating sites were included to mitigate the selection bias.

This observational study was conducted in compliance with the International Ethical Guidelines for Biomedical Research Involving Human Subjects, Good Clinical Practice guidelines, and the Declaration of Helsinki, 1964; local laws; SAS order 3470/2009; and, Organic Law 3/2018, of December 5, on the Protection of Personal Data and guarantee of digital rights. This study received an IEC approval by the Ethics Committee of Hospital Universitario Ramón y Cajal (25/03/2021 ACTA 410). The patients signed an informed consent form prior to data collection. Considering that the research could not be carried out without the omission of written consent patients who died to avoid bias, the local Ethics Committee waived the requirement of written authorisation (as established by local Spanish regulations: RD 957/2020) in these cases. This study was registered with the Spanish Registry of Clinical Studies (Ref: 0004-2021-OBS) in May 2021.

### 2.2 Participants

The study included adult patients treated with 450 mg of encorafenib once daily and 45 mg of binimetinib twice per day orally, according to the current summary of product characteristics (SmPC), in the 1^st^ line or after progression to a 1^st^ line with immune checkpoint inhibitors in the unresectable or metastatic setting, starting this treatment at least 6 months before the patient’s inclusion in the study. Previous BRAF and/or MEK inhibitors were prohibited, except in the adjuvant setting if it ended ≥6 months before the initiation of the studied line of treatment in advanced/metastatic setting. Patients with previous systemic treatments, including chemotherapy or other treatments, other than a single 1^st^ line ICIs in an advanced setting were excluded. Patients with any contraindications to receiving encorafenib or binimetinib, those currently participating in interventional studies, and those having any tumour other than melanoma with active treatment were excluded.

### 2.3 Objectives and outcomes

This study aimed to determine the effectiveness and safety of encorafenib plus binimetinib administration in the treatment of unresectable or metastatic BRAF^V600^-mutant melanoma in the real-world. The objectives of this study were to describe the baseline demographic and pathological characteristics of the population of patients treated with encorafenib plus binimetinib in Spain, treatment compliance, effectiveness and safety, survival outcomes and the influence of prognostic factors and prior systemic treatments on effectiveness.

Efficacy endpoints included objective response rate (ORR), defined as the percentage of patients that achieve partial response (PR; >30% decrease in the sum of target lesions, considering the baseline sum diameter as reference) or complete response (CR; disappearance of all target lesions) as their best responses; duration of response (DoR), defined as the time elapsed from the first determination of response (PR or CR) to the progression of the disease or death, whichever occurs first, in patients with response; disease control rate (DCR), defined as the percentage of patients with CR, PR or stable disease (SD) as their best response; duration of disease control (DoDC), calculated among responders and stable patients from the date of the start of treatment until the documentation of progression or death; progression-free survival (PFS) rate at 12 months, defined as the percentage of patients alive and free of progression (increase of >20% in the sum of diameters of target lesions, considering the smallest sum on study as the reference) at 12 months after the start of treatment; median PFS, defined as the time elapsed from the start of treatment until progression or death, whichever occurs first; and, OS, calculated from the start of treatment until the date of death due to any cause. For PFS, patients who were lost to follow-up or had no event of progression or death by the time of the analysis were censored at the date of the last tumour assessment or last contact when there was no progression, whichever occurred last. For OS, patients who were alive at the time of analysis were censored at their last follow-up. Safety endpoints included adverse events classified according to severity, number of interruptions, dose adjustments, and permanent treatment discontinuation.

### 2.4 Statistical analysis

The initially planned sample size was at least 50 patients, assuming that it was adequate for a descriptive study. The number of participation sites was selected considering adequate geographic coverage and the involvement of different centre types. After achieving the expected sample size, the study was amended to include up to 100 patients. The study was descriptive and no formal assumptions on safety or efficacy endpoints were made for the sample size calculations.

Statistical analyses were performed using descriptive statistics for quantitative variables and frequency, percentages and confidence intervals for categorical variables. Time-to-event endpoints and survival analyses were performed using the Kaplan-Meier method and risk factor analyses were performed based on the Cox proportional hazard model. All statistical tests were two-tailed and results with p<0.05 were considered significant. All statistical analyses were performed using R (version 3.6.3 [2020-02-29] “Holding the Windsock” R Foundation for Statistical Computing, Vienna, Austria) and SPSS (IBM SPSS Statistics version 26, Armonk, NY, US). Figures and tables were generated using RStudio (version 1.2.5033 2009-2019 RStudio, Inc., Boston, MA, US).

## 3 Results

### 3.1 Baseline characteristics

Between September 2021 and March 2023, 117 patients with unresectable or metastatic BRAF^V600^-mutant melanoma were enrolled. The baseline characteristics of the enrolled patients are summarised in **Table 1**. The median age was 59 years (range, 23-89 years) and the histological subtypes were superficial extension (51 patients, 43.6%), nodular (42 patients, 35.9%), acral (three patients, 2.6%) and unknown (21 patients, 17.9%). Metastasis was present at treatment initiation in 116 (99.1%) patients, with more than three metastatic sites in 49 (41.9%) patients. The most frequent metastatic locations were nodes (59.0%), lungs (43.6%), and cutaneous (26.5%). Brain and liver metastases were observed in 25 (21.4%) and 24 (20.5%) patients, respectively. Lactate dehydrogenase (LDH) levels were increased in 35.9% of patients. Encorafenib plus binimetinib was the 1^st^ line of treatment for 89 patients (76.1%) and 2^nd^ line of treatment after previous treatment with ICI in 28 patients (23.9%). Among patients included in the second line treatment, 24 patients (85.7%) had progressed to ICI during treatment and 4 (14.3%) after discontinuation of ICI treatment. ICIs comprised treatment with a single ICI such as nivolumab (11 patients), or pembrolizumab (nine patients); dual ICIs such as nivolumab plus ipilimumab (4 patients); clinical trial-related ICI (three patients) and ICI treatment not specified (one patient) (**Supplementary Figure 1**).

**Table 1:** Baseline patient characteristics for the patients enrolled in BECARE observational study, the COLUMBUS study, that led to approval of EB.

Characteristics; unit	BECARE n = 117	COLUMBUS trial n = 192
Median age (range); years	59 (23-89)	57 (20-89)
Sex, n (%)		
Male	70 (59.8)	115 (59.9)
*BRAF^V600^ mutational status, n (%)*		
V600E	75 (64.1)	170 (88.5)
V600K	10 (8.5)	22 (11.5)
V600R	5 (4.3)	–
V600 not specified	37 (31.6)	–
ECOG PS; n (%)		
0	75 (64.1)	136 (70.8)
1	25 (21.4)	56 (29.2)
2	9 (7.7)	-
3	3 (2.6)	-
UK	5 (4.3)	-
Stage at inclusion AJCC*; n (%)		
IIIC	1 (0.9)	–
IV (M1a)	29 (24.8)	26 (13.5)
IV (M1b)	22 (18.8)	34 (17.7)
IV (M1c)	38 (32.5)	123 (64.1)
IV (M1d)	26 (22.2)	–
Number of metastatic locations; n (%)		
1	38 (32.5)	47 (24.5)
2	30 (25.6)	58 (30.2)
≥3	49 (41.9)	87 (45.3)
Metastatic locations; n (%)		
Nodes	69 (59)	-
Lung	51 (43.6)	-
Bone	30 (25.6)	-
Cutaneous	31 (26.5)	-
Liver	24 (20.5)	-
Brain	25 (21.4)	9 (4.7)
LDH levels; n (%)		
Normal	70 (59.8)	137 (71.4)
> 1 - ≤ 2 ULN	26 (22.2)	-
> 2 ULN	16 (13.7)	-
UK	5 (4.3)	-

AJCC, American Joint Committee on Cancer; ECOG PS, Eastern Cooperative Oncology Group performance status; LDH, Lactate Dehydrogenase; UK, unknown; ULN, upper limit normal. *AJCC 8^th^ edition was used in the BECARE study and AJCC 7^th^ edition in the COLUMBUS trial.

### 3.2 Treatment compliance

The median duration of treatment with encorafenib plus binimetinib was 11.5 months (95% CI, 8.5-13.5). At the data cut off date, 80 patients (68.4%) had permanently discontinued treatment owing to progression (58.1%), exitus (6.8%), unacceptable toxicity (6.8%), clinical deterioration (1.7%), loss to follow-up (1.7%), complete response (1.7%) and patient’s decision (0.9%). Encorafenib plus binimetinib treatment was temporarily discontinued in 37 (31.6%) patients, due to toxicity in 27 (23.1%); physician criteria, three (2.6%); requirement of radiotherapy, three (2.6%); management of coronavirus disease (COVID-19), three (2.6%); and, surgical intervention, 1 (0.9%). Dose reduction was required in 29 (24.8%) patients to manage treatment-related adverse events. The encorafenib dose was reduced to daily doses of 300 mg in 20 (17.1%) patients, 200 mg in six (5.1%), and 100 mg in one (0.9%). Binimetinib dose was reduced to two daily doses of 30 mg in 23 (19.7%) patients. Two and six patients did not report the details of dose reductions of encorafenib and binimetinib, respectively. Both treatment doses were reduced in 20 (17.1%) patients.

### 3.3 Efficacy

**Figure 1:**
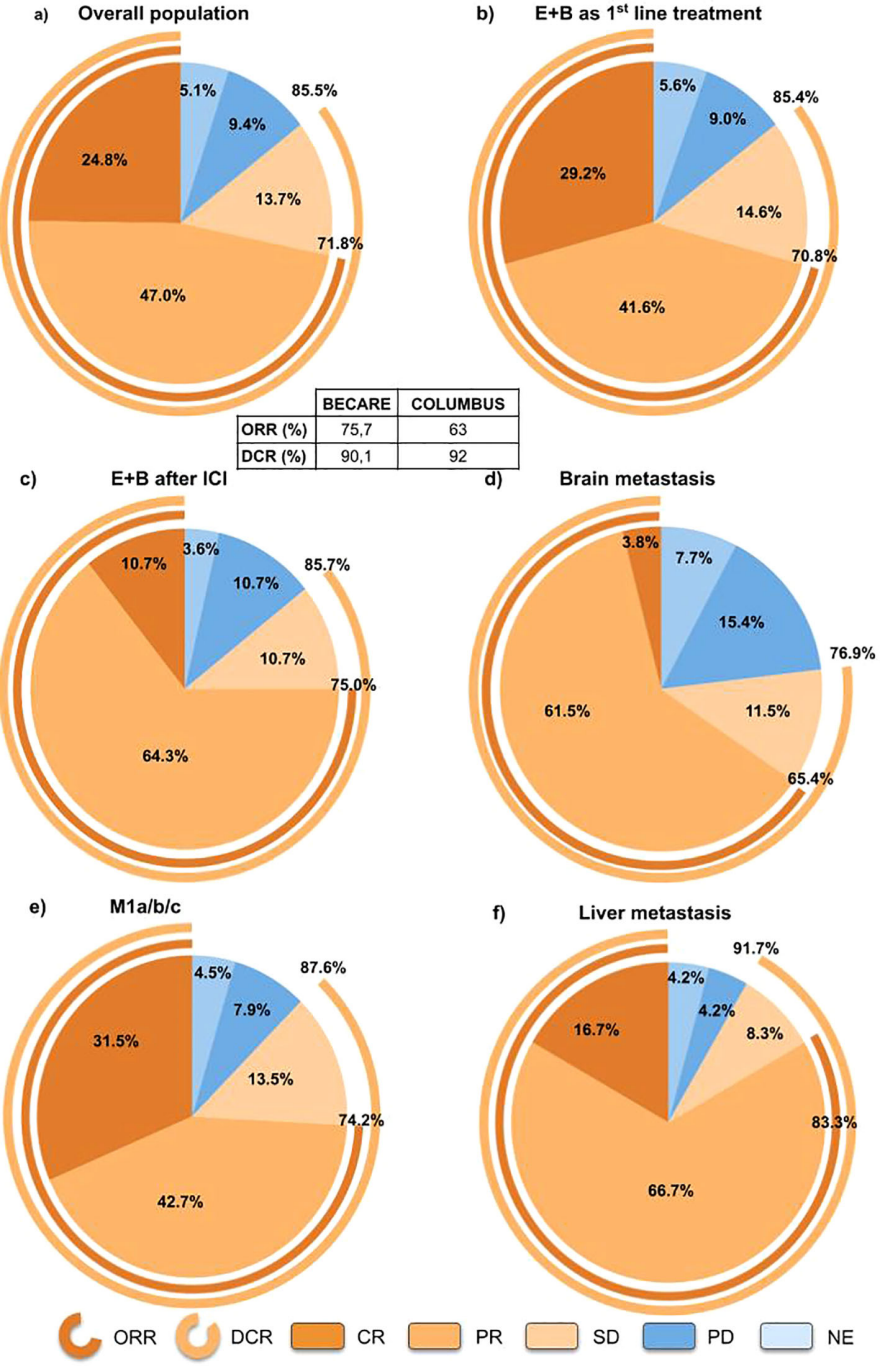
Best overall response for **(A)** overall population, **(B)** patients treated with encorafenib+binimetinib as 1^st^ line, **(C)** patients treated with encorafenib+binimetinib as 2^nd^ line, **(D)** patients with brain metastasis, **(E)** patients with liver metastasis, and **(F)** patients with M1a/b/c melanoma. DCR, disease control rate; E+B, encorafenib plus binimetinib; ICI, Immune checkpoint inhibitors; ORR, objective response rate; NE, not evaluable.

With a median follow-up of 13.8 months (95% CI, 12.0-17.4), the ORR was 75.7% and the DCR was 90.1% (**Figure 1A**). The median follow-up was 14.6 months (95% CI: 12.0-19.0) and 12.7 months (95% CI: 9.9, 17.3) for patients in the 1^st^ and 2^nd^ line treatment, respectively. The ORR and DCR for patients treated with encorafenib plus binimetinib as 1^st^ line treatment were 75% and 90.5%, respectively, whereas the ORR and DCR for patients treated in the 2^nd^ line were 77.8% and 88.9%, respectively (**Figures 1B, C**). The DoDC was 9.7 months (95% CI 6.9-16.9) for the overall population; 9.9 months (95% CI 7.8-18.3), patients treated with encorafenib plus binimetinib as 1^st^ line treatment; and, 6.8 months (95% CI 3.3-not reached [NR]), patients treated after immune checkpoint inhibitors (**Supplementary Figure 2**). Patients with brain metastasis (n=25) showed ORR and DCR of 70.8% and 83.3%, respectively, and patients with M1a/b/c showed ORR and DCR of 74.2% and 87.6%, respectively (**Figures 1D, F**). Patients with liver metastasis had an ORR and DCR of 83.3% and 91.7% **Figure 1E**. The ORR was 81.9% and 56.5% in patients with ECOG 0 and ≥1, respectively.

**Figure 2:**
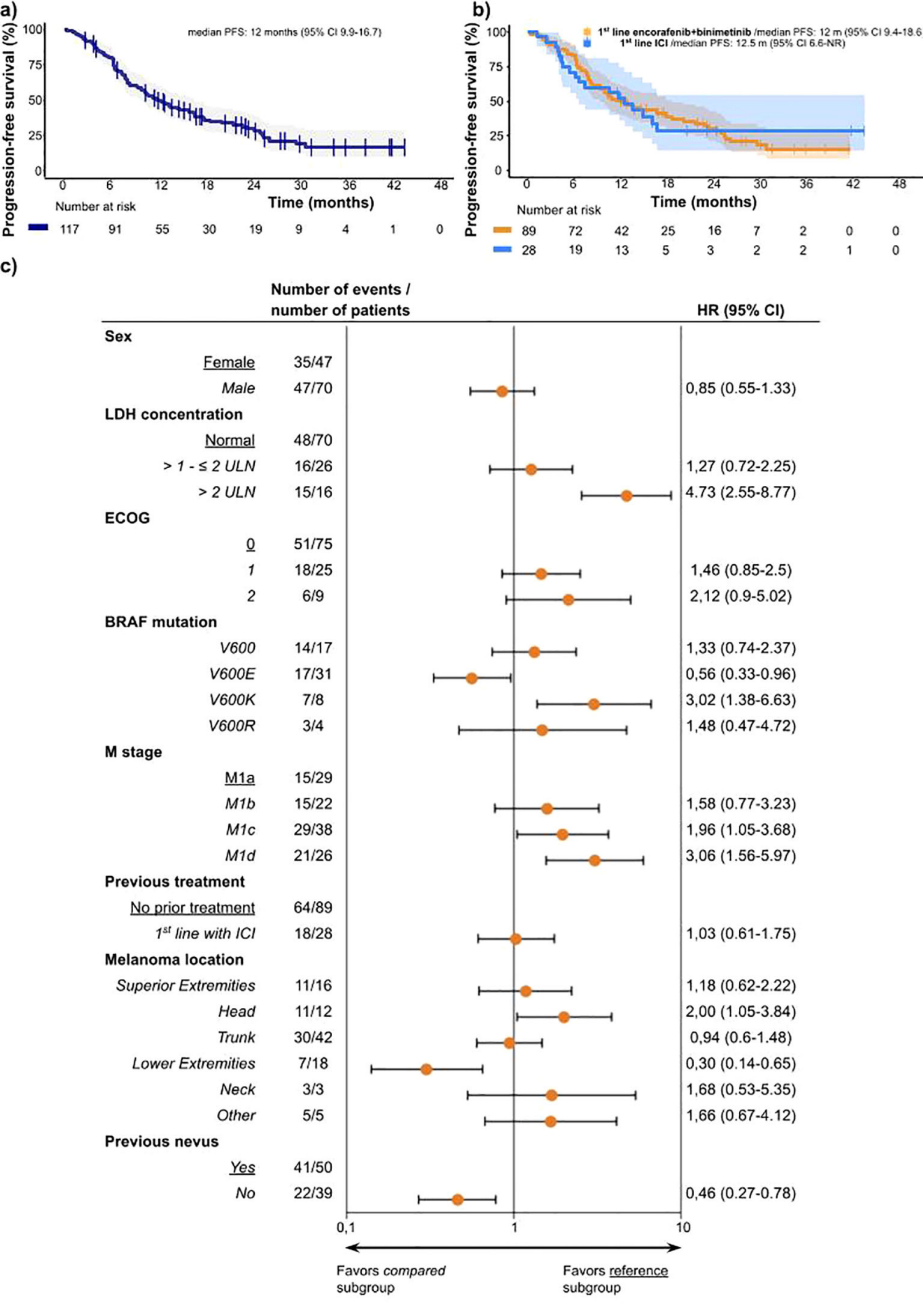
**(A)** Kaplan-Meier curves of progression-free survival for overall population and median PFS. **(B)** Kaplan-Meier curves of progression-free survival and median PFS for encorafenib plus binimetinib as 1^st^ line treatment (orange) and encorafenib plus binimetinib after treatment with checkpoint inhibitors (blue). **(C)** Progression-free survival by subgroups according to baseline characteristics. For each characteristic, the subgroups are compared with other subgroup as reference. This reference is listed first and underlined and has no Hazard ratio calculated, except for BRAF mutation and Melanoma location, which were compared with the opposite option. As an example, BRAF^V600E^ mutation was compared to population not having BRAF ^V600E^ mutation. Compared groups are written in *italic*. ECOG status 3, Melanoma location Hand and Melanoma location Feet are not included due to the very low number of cases and all this patient did not survive. ECOG PS, Eastern Cooperative Oncology Group performance status; LDH, Lactate Dehydrogenase; ULN, upper limit normal.

The median global PFS was 12.0 months (95% CI, 9.9-16.7) and the 12-month PFS rate was 50.7% (95% CI, 42.3-60.8) (**Figure 2A**). Efficacy was similar in 1^st^ and 2^nd^ line, with a median PFS and 12-month PFS of 12.0 months (95% CI, 9.4-18.6) and 50.3% (95% CI, 40.7-62.0) for 1^st^ line treatment, and 12.5 months (95% CI, 6.6-NR) and 52% (95% CI, 36.1-74.8) for 2^nd^ line treatment, respectively (**Figure 2B**, **Supplementary Figure 3**). The median PFS in patients with ECOG 0, 1 and ≥2 was 16.0 months (95% CI, 11.4-22.8), 7.7 months (95% CI, 5.1-29.6) and 6.6 months (95% CI, 6.3-NR), respectively (**Supplementary Figure 4**). For patients with M1a/b/c melanoma, the median PFS was 13.5 months (95% CI, 10.7-21.6).

Receiving encorafenib plus binimetinib treatment as 1^st^ or 2^nd^ line treatment did not significantly affect the risk of progression or death (HR 1.03, 95% CI: 0.61-1.75; p=0.900) (**Figure 2C**). Male patients had a similar risk of progression as that of female patients (HR 0.85, 95% CI: 0.55-1.33; p=0.483). Analysis of histology and location as prognostic factors showed a lower risk of progression or death for melanoma located at lower extremities (HR 0.30; 95% CI: 0.14-0.65; p=0.002) and higher risk when located in the head (HR 2.00, 95%CI: 1.05-3.84; p=0.036) (**Figure 2C**). The univariate Cox analysis of baseline BRAF^V600^ mutations showed that the type of mutation correlates with the prognosis, showing a lower risk of progression or death for BRAF^V600E^ (HR 0.56, 95%CI: 0.33-0.96; p=0.035) and higher risk for BRAF^V600K^ mutation (HR 3.02, 95%CI: 1.38-6.63; p=0.006). The patients with normal levels of LDH and elevated >1 and ≤2 times above (upper limit of normal (ULN) had comparable PFS, but the risk was higher in patients with LDH levels elevated >2 times above ULN (HR 4.73, 95%CI: 2.55-8.77; p<0.001) (**Figure 2C**).

Subgroup analyses of the M subtypes showed a worse prognosis in subtypes M1d (HR 3.06, 95% CI: 1.56-5.97; p=0.001) and M1c (HR 1.96, 95% CI: 1.05-3.68; p=0.035) compared to those in M1a and M1b (HR 1.58, 95% CI: 0.77-3.23; p=0.214). The median PFS for the M1d subgroup was 6.3 (95% CI, 6.12-10.25) months and the 12-month PFS rate was 25.2% (**Supplementary Figure 5**). Patients with M1a/b/c had a median OS of 25.8 months (95% CI, 17.9-NR) (**Supplementary Figure 6**). Patients with liver metastasis had a median PFS of 11.4 (95% CI, 6.3-25) months and median OS of 14.8 (95% CI, 7.4-NR) months (**Supplementary Figure 7**). The OS rate at 12 months in patients with brain metastasis was 35.2% (95% CI, 20.4-60.7). Only one patient with brain metastasis was included after progression to ICI, reporting PR as the best response and being alive and free of progression 14 months after the initiation of encorafenib plus binimetinib treatment.

**Figure 3:**
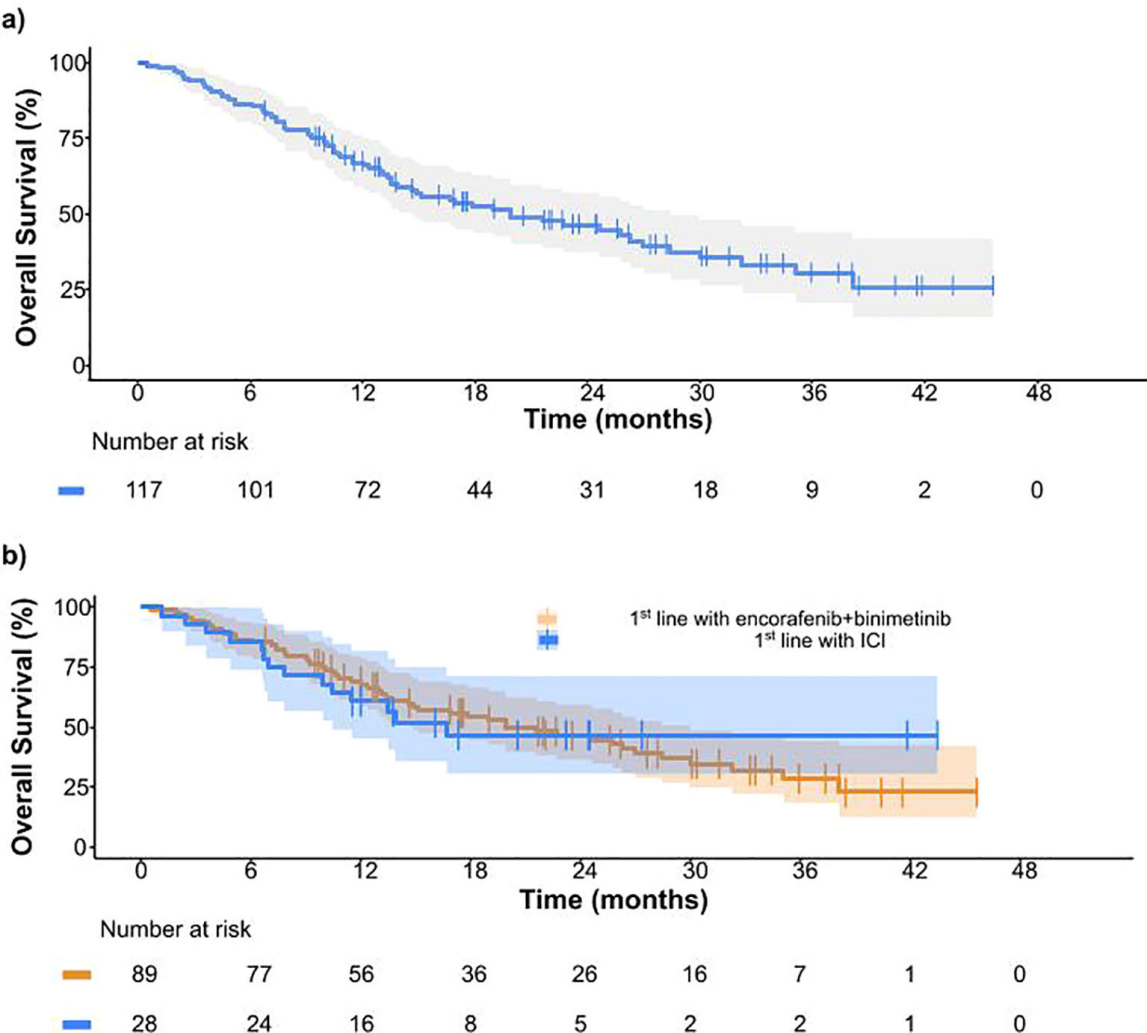
**(A)** Overall survival in all patients from BECARE study. **(B)** Overall survival for patients receiving encorafenib plus binimetinib as 1^st^ line treatment (orange) and encorafenib plus binimetinib after treatment with checkpoint inhibitors (blue).

For patients receiving encorafenib plus binimetinib as 1^st^ line and 2^nd^ line treatment, the 12-month OS rates were 69.1% and 60.7%, respectively (**Figure 3**).

### 3.4 Safety

**Figure 4:**
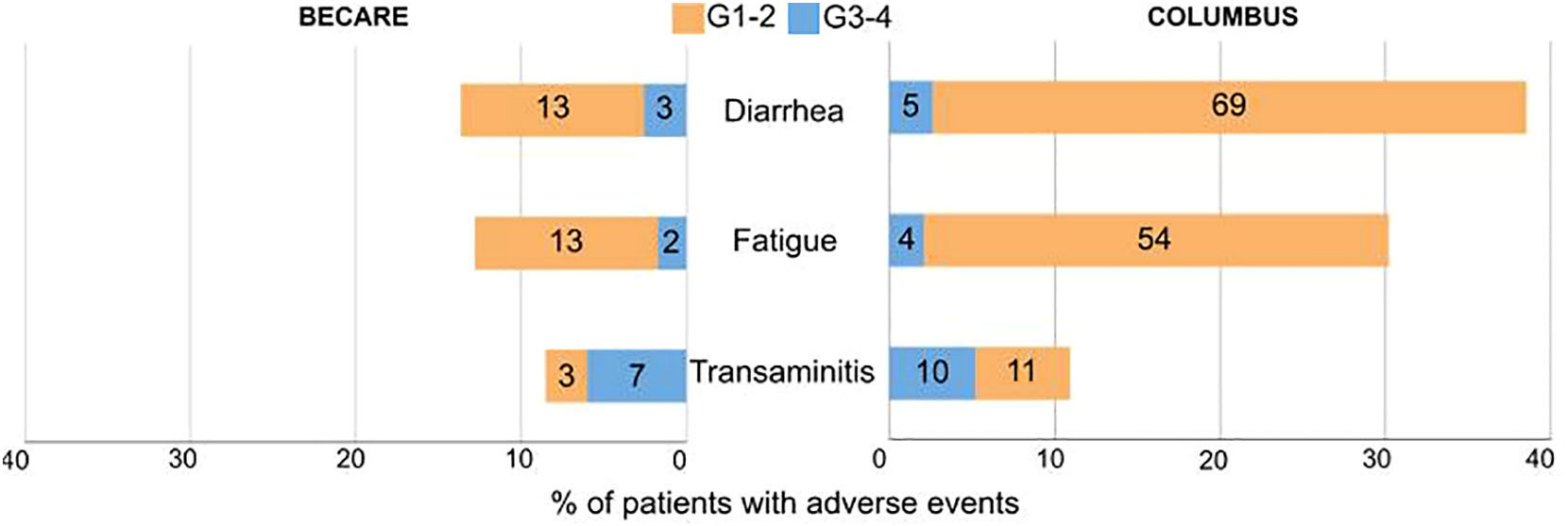
Bar chart of most frequent adverse events with 5% threshold in BECARE (left) and COLUMBUS (right) study. The number of patients with the AE are indicated in each bar.

Of the 117 patients, 50 (42.7%) experienced adverse events, 47 (40.2%) of which had AE related to encorafenib and binimetinib. The most frequent treatment-related adverse events were diarrhoea (n=16, 13.7%), fatigue (n=12, 10.3%) and transaminitis (n=10, 8.5%) (**Supplementary Tables 1**, **2**). Grade 3-4 treatment-related adverse events were reported in 17 patients (14.5%), and the most frequent were transaminitis in seven (6%) patients; diarrhoea, three (2.6%); and, fatigue, three (2.6%) (**Figure 4**). No grade 5 toxicities were observed. The safety profile was not significantly different between patients receiving encorafenib plus binimetinib as 1^st^ line and those treated after ICI.

## 4 Discussion

BECARE is the 1^st^ observational study to report the real-world effectiveness and safety of encorafenib plus binimetinib for the treatment of unresectable or metastatic BRAF^V600^-mutant malignant melanoma in Spain.

The PFS reported in the BECARE trial was equivalent to the reported in the phase III registration study COLUMBUS, reaching a median PFS of 12 vs 14.9 months and a 12-month PFS rate of 50.7% vs. 56.2% for BECARE and COLUMBUS, respectively. The PFS values were slightly lower in the BECARE study, which was potentially influenced by the fact that the real-world population had worse prognostic factors (i.e. older population [59 vs 57], higher rate of brain metastasis [21.4% vs 4.7%], or a higher % of patients with LDH above the ULN [47.7% vs 28.6%]). This finding is consistent with those of previous reports, suggesting that patients with brain metastasis or high LDH levels are underrepresented in phase III clinical trials. The median PFS was similar to that reported for dabrafenib plus trametinib (11.4 months, COMBI-v study) (10) and higher than that for cobimetinib plus vemurafenib (9.9 months) (4) in phase III clinical trials. The DCR was similar for all TKIs, and the ORR reported in the BECARE trial (75.7%) was higher than that in the COLUMBUS study (63%) and phase III trials with other BRAF/MEK inhibitors such as dabrafenib plus trametinib or cobimetinib plus vemurafenib (5, 11).

A subgroup analysis was performed to better understand the efficacy of encorafenib plus binimetinib in specific populations. Consistent with previous reports (12), our data showed that patients with M1c metastasis (distant metastasis to non-CNS visceral sites) had a higher risk of disease progression or death (HR, 1.96) than those with M1a metastasis. Additionally, our study included 26 (22.2%) patients with M1d (distant metastasis in the CNS) and had an even worse prognosis, with an HR of 3.06 compared to M1a. The COLUMBUS study only included nine (4.7%) patients, and the coBRIM study only included one (0.4%) patient with brain metastasis. These patients had a slightly lower response rate and the median PFS was nearly half that of the overall population. However, the median PFS in patients with brain metastasis was higher than that in previous reports (12–14), suggesting that encorafenib plus binimetinib is a feasible option for these patients.

With regard to patients receiving encorafenib plus binimetinib as 2^nd^ line after ICI, in the BECARE study, the ORR, DCR, and PFS outcomes were similar to those in patients treated with the combination in the 1^st^ line. Although the numerical 12-month OS rate was slightly lower than that in the 1^st^ line, the difference was not statistically significant. Previous real-world studies with other TKIs have also shown similar trends (15). Thus, our results show that encorafenib plus binimetinib is a feasible treatment option even after progression to ICI. Regarding this observation, early evidence on sequentiality indicated that ICI first could reach better outcomes than BRAF/MEK inhibitors in advanced melanoma (16). However, the study was not designed to compare encorafenib plus binimetinib with ICI, and therefore we could not provide recommendations on sequentiality, which may require further evidence from randomized trials or real world evidence using propensity score in bigger patient datasets.

The toxicity profile in the real world is manageable and validated the results of the phase III clinical trial. According to our results, in the real world, treatment-related adverse events were lower than those in the registration phase III COLUMBUS clinical trial, which led to the approval of encorafenib plus binimetinib treatment by the FDA and EMA. The number of AEs was lower in our study. Most common G3-4 AE proportions were similar between the BECARE and COLUMBUS studies, with proportions of 2.6% vs. 2.6%, 1.7% vs. 2.1%, and 4.3% vs. 5.2% for diarrhoea, fatigue, and transaminitis respectively.

A decrease in the number of adverse events resulted in fewer dose reductions (24.8% vs. 48%) and lower discontinuation rates (6.0% vs. 8.3%). These results are consistent with previous observational studies (17) and support the idea that the implemented management strategies in clinical practice and the experience of handling treatment-related adverse events contributed to reducing the number of discontinuations due to toxicity (18). The low discontinuation rates observed might respond to a less stringent discontinuation criteria in the real-world than in clinical trials, and the potential bias due to the inherently less stringent monitoring of adverse events in observational studies can not be discarded.

Discontinuation rates due to toxicity after treatment with other TKIs were 14% for cobimetinib plus vemurafenib (4), 7% for vemurafenib (4) and 13% for dabrafenib plus trametinib (10). For immunotherapy, toxicity-related discontinuation in a real-world context has been reported to range from 22 to 32% (19, 20). Discontinuation of melanoma treatment due to toxicity in a real-world context has been reported to range from 4 to 10% for targeted therapies (14, 15, 18, 21). Consistent with our results, toxicities appear to be better managed in clinical practice leading to fewer toxicity-related discontinuations.

The limitations of this study are those inherent to retrospective observational studies, such as limited follow-up, a higher missing data rate, lack of standardised tumour assessment criteria (i.e. RECIST) and small subgroups. Nevertheless, most data was available to ensure reliable conclusions. This study also had no control arm to contextualise the results. Although follow-up was acceptable to address the proposed survival outcomes, further follow-up after long term will help to confirm the benefit of E+B on survival.

In conclusion, encorafenib plus binimetinib showed comparable efficacy than that in the phase III registration trial and good tolerability, despite worse prognosis in the real-world population studied, which comprised more cases of brain and liver metastases. Encorafenib plus binimetinib is a feasible treatment option for unresectable and metastatic melanoma in the real-world, with similar outcomes as 1^st^ line treatment or after treatment with ICI, including in patients with brain metastasis.

## Data Availability

The raw data supporting the conclusions of this article will be made available by the authors, without undue reservation.
