# Supplementary material for: Real-life evidence of encorafenib plus binimetinib in patients with unresectable advanced or metastatic BRAFV600-mutant melanoma in Spain: the BECARE (GEM-2002) trial
Source: Front Oncol. 2025 Feb 26;15:1466185. doi: 10.3389/fonc.2025.1466185 (PMC11897558; doi:10.3389/fonc.2025.1466185)
Supplement: Supplementary file 1 [file DataSheet1.pdf]

Supplementary information.

Supplementary Figure 1. Patient flowchart. Abbreviations: EB: encorafenib plus binimetinib; CR: Complete Response; PD: Progress Disease; PI: Principal Investigator.

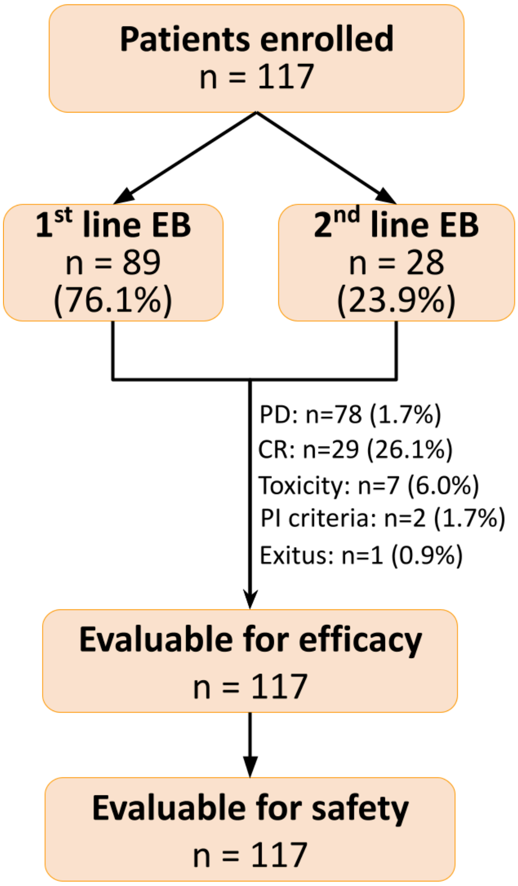

Supplementary Figure 2. Swimmer plot showing the date of response, progression, or death and the start of systemic treatments after encorafenib plus binimetinib for patients treated with encorafenib plus binimetinib as 1st line (**up**) and after treatment with immune checkpoint inhibitors (**down**).

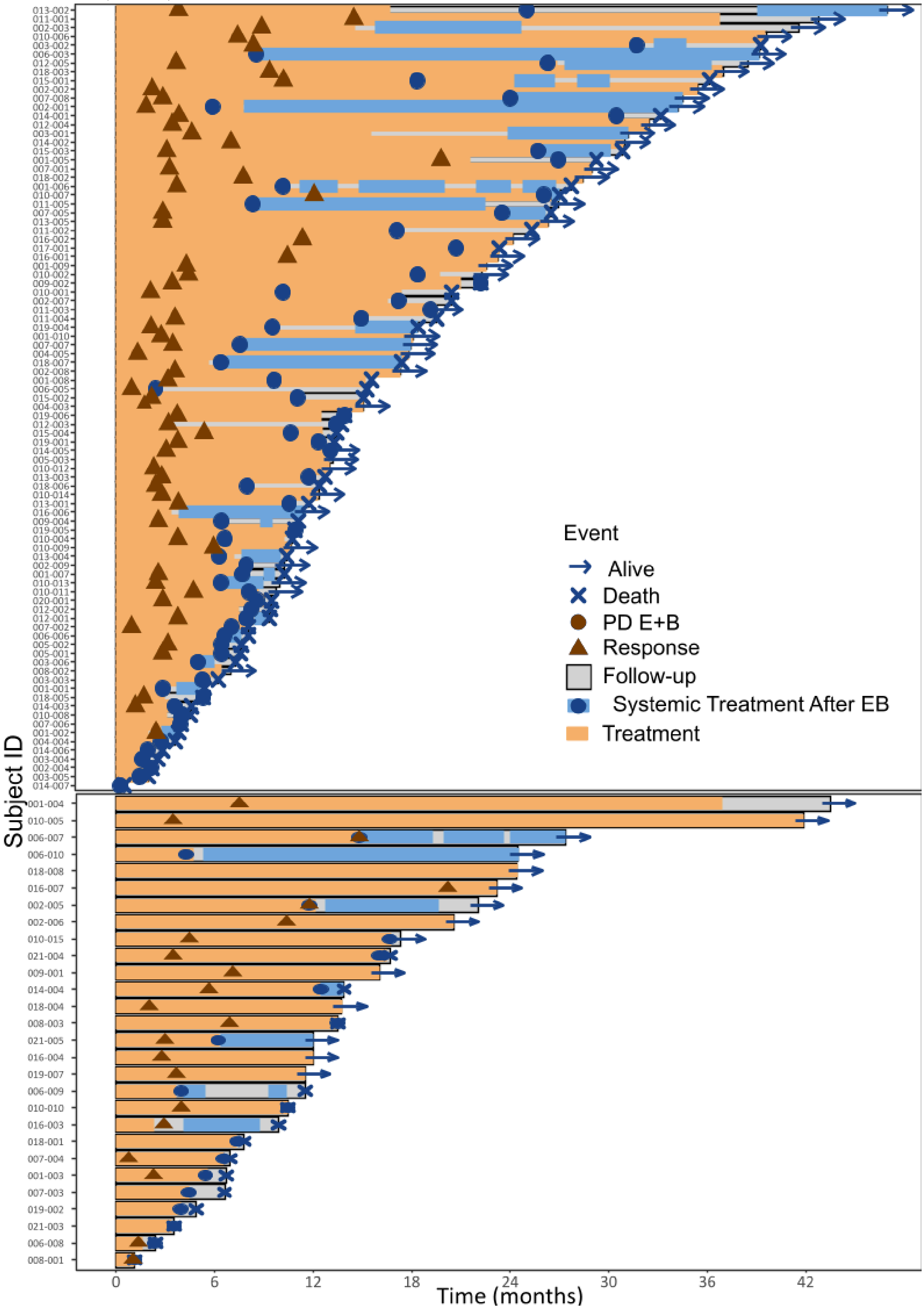

Supplementary Figure 3. a) Progression-free survival by subgroups according to baseline characteristics for patients treated with first line encorafenib plus binimetinib. b) Progression-free survival by subgroups according to baseline characteristics for patients treated with second line encorafenib plus binimetinib after ICI. For each characteristic, the subgroups are compared with other subgroup as reference. This reference is listed first and <u>underlined</u>. Compared groups are written in *italic*. Abbreviations: ECOG PS: Eastern Cooperative Oncology Group performance status; LDH: Lactate Dehydrogenase; ULN: upper limit normal.

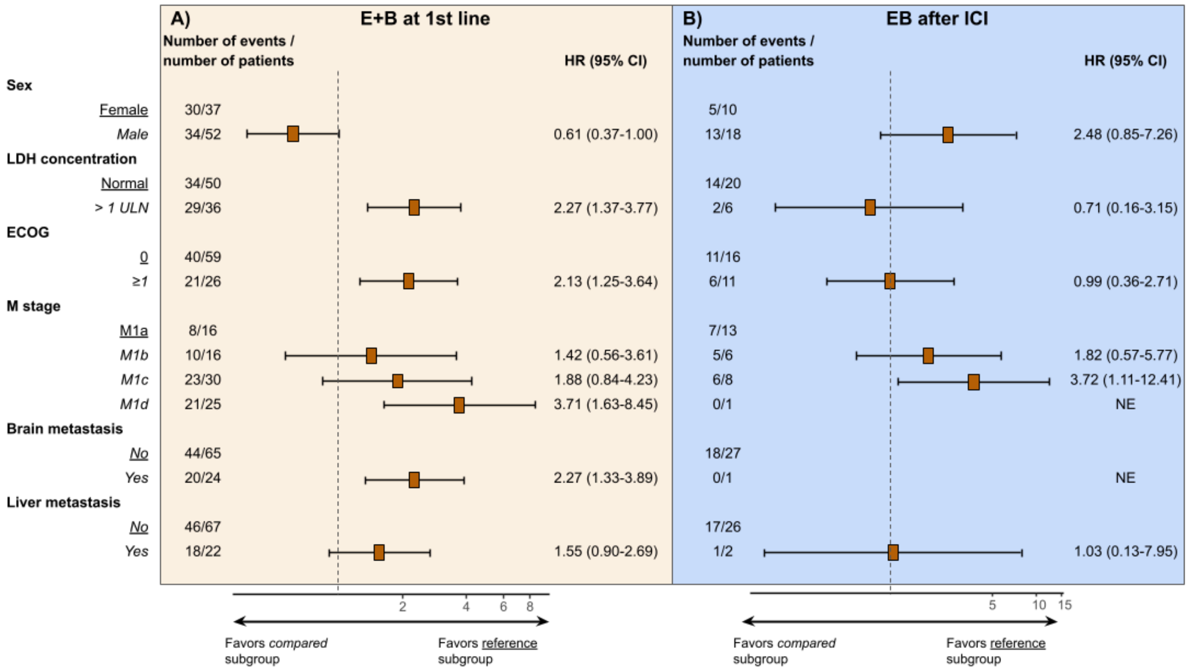

Supplementary Figure 4. a) Kaplan-Meier curve of progression-free survival for encorafenib plus binimetinib in patients according to ECOG PS. **b)** Kaplan-Meier curve of overall survival for encorafenib plus binimetinib in patients according to ECOG PS.

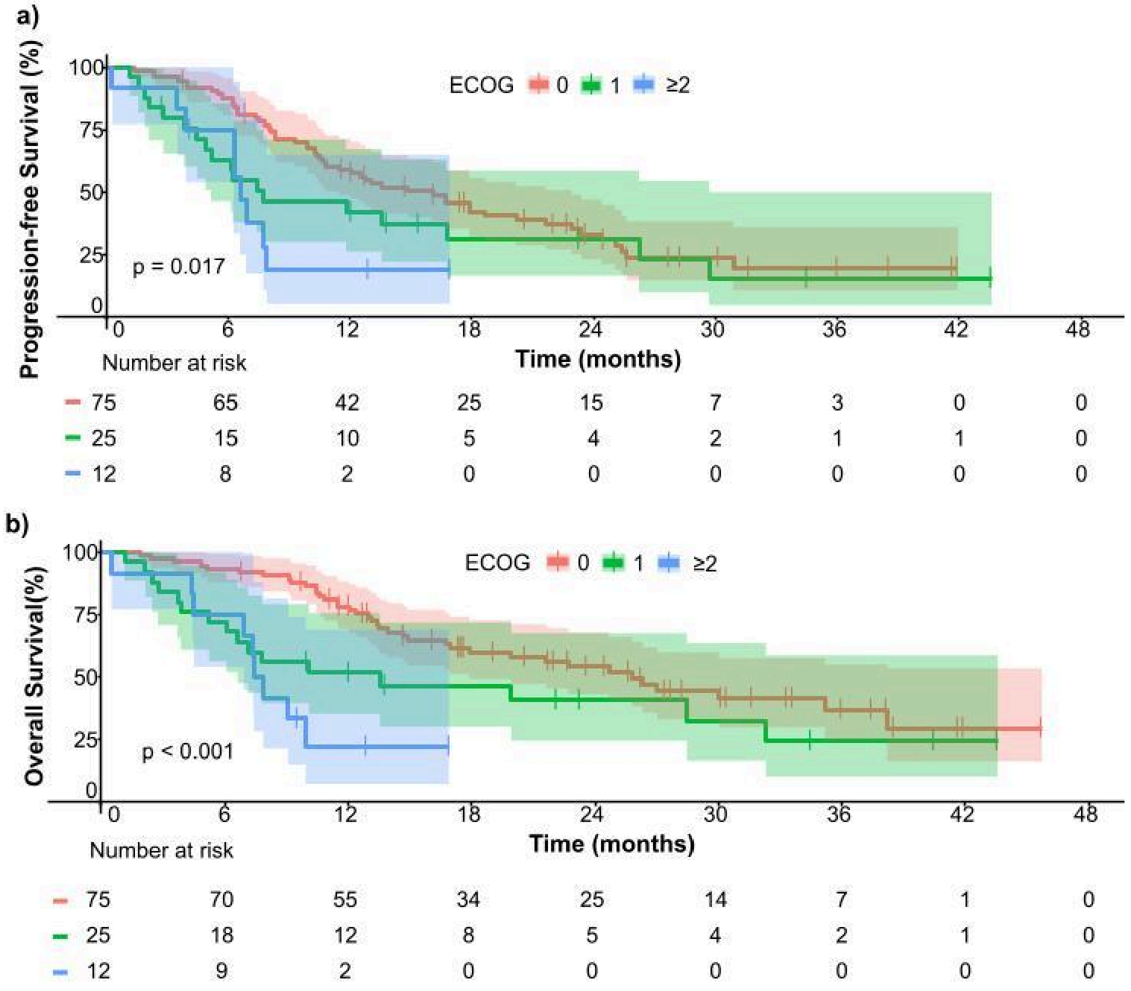

Supplementary Figure 5. a) Kaplan-Meier curve of progression-free survival for encorafenib plus binimetinib in patients with brain metastasis. **b)** Kaplan-Meier curve of overall survival for encorafenib plus binimetinib in patients with brain metastasis.

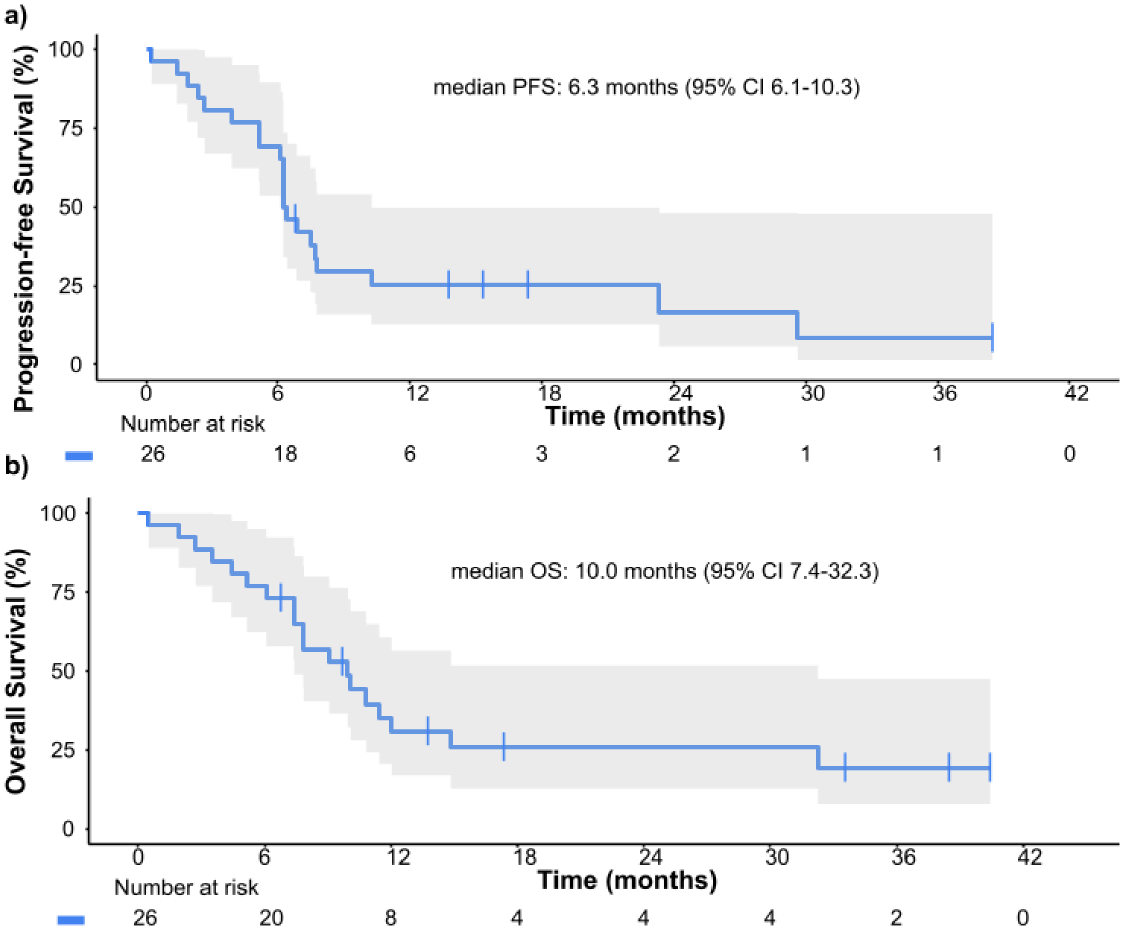

Supplementary Figure 6. a) Kaplan-Meier curve of progression-free survival for encorafenib plus binimetinib in patients with M1a/b/c. **b)** Kaplan-Meier curve of overall survival for encorafenib plus binimetinib in patients with M1a/b/c. Abbreviations: NR: Not reached.

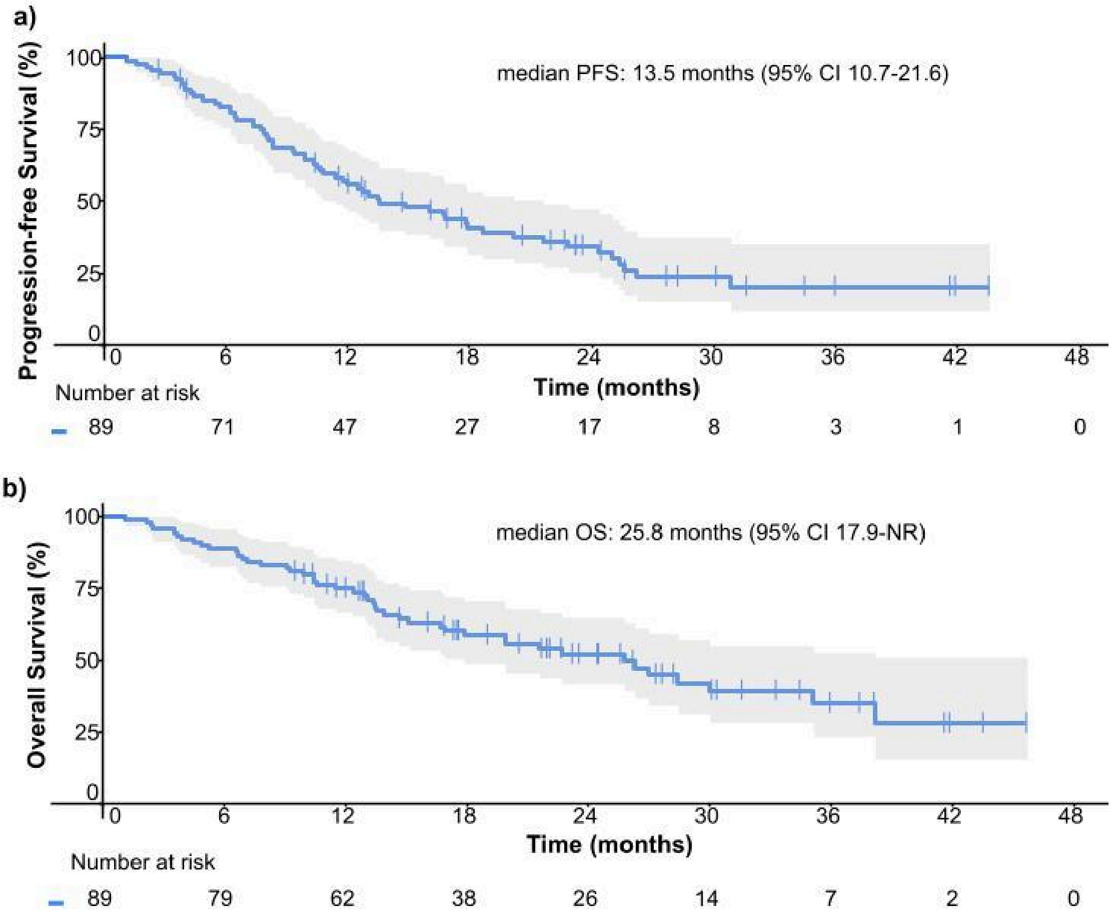

Supplementary Figure 7. a) Kaplan-Meier curve of progression-free survival for encorafenib plus binimetinib in patients with liver metastasis. **b)** Kaplan-Meier curve of overall survival for encorafenib plus binimetinib in patients with liver metastasis. Abbreviations: NR: Not reached.

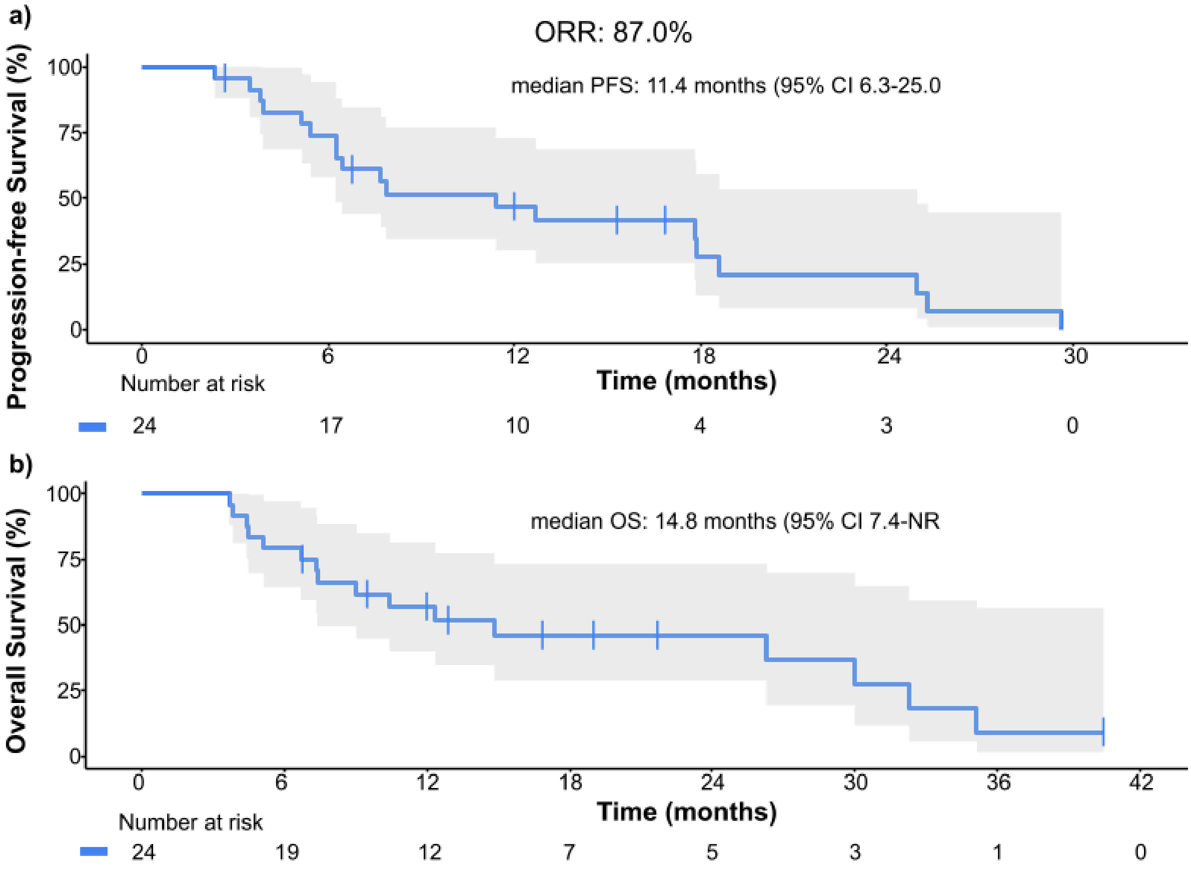

Supplementary Table 1. Grade of most frequent treatment-related adverse events (TRAEs) in BECARE study detected in patients treated with encorafenib plus binimetinib in 1st line, in 2nd line after ICI and for the overall population.

TRAEs	E+B in 1st line		E+B after ICI		Overall	
Event, n (%)	G1-2	G3-4	G1-2	G3-4	G1-2	G3-4
Diarrhoea	11 (12.4)	1 (1.1)	2 (7.1)	2 (7.1)	13 (11.1)	3 (2.6)
Fatigue	8 (12.4)	2 (2.2)	2 (7.1)	0 (0.0)	10 (8.5)	2 (1.7)
Transaminitis	3 (3.4)	8 (9.0)	0 (0.0)	2 (7.1)	3 (2.6)	10 (8.5)

Supplementary Table 2. List of all treatment-related adverse events and their grade and intensity.

Patient Number	AE CTCAE	AE Grade	AE SAE	AE Intensity	AE Related to
001-002	Colonic ulcer	4	Yes	Hospitalisation	Binimetinib Encorafenib
001-005	Colonic ulcer	1	No	Unknown	Binimetinib Encorafenib
001-006	Alanine aminotransferase increased	1	No	Unknown	Binimetinib Encorafenib
001-006	Arthralgia	1	No	Unknown	Binimetinib Encorafenib
001-006	Nausea	1	No	Unknown	Binimetinib Encorafenib
001-006	Alanine aminotransferase increased	2	No	Unknown	Binimetinib Encorafenib
002-003	Retinal vascular disorder	3	Yes	Significant medical event	Binimetinib
002-003	Arthralgia	3	Yes	Significant medical event	Encorafenib
003-001	Uveitis	2	No	Unknown	Binimetinib Encorafenib
003-001	Diarrhoea	1	No	Unknown	Binimetinib Encorafenib
003-001	Fatigue	1	No	Unknown	Binimetinib Encorafenib
003-001	Fatigue	1	No	Unknown	Binimetinib Encorafenib
003-001	Constipation	2	No	Unknown	Binimetinib Encorafenib
003-001	Constipation	2	No	Unknown	Binimetinib Encorafenib
003-001	Constipation	1	No	Unknown	Binimetinib Encorafenib
003-001	Arthralgia	UK	No	Unknown	Binimetinib Encorafenib
003-001	Rash maculo-papular	2	No	Unknown	Binimetinib Encorafenib
003-001	Constipation	1	No	Unknown	Binimetinib Encorafenib
003-002	Constipation	1	No	Unknown	Binimetinib Encorafenib
003-002	Myalgia	1	No	Unknown	Binimetinib Encorafenib
003-002	Diarrhoea	1	No	Unknown	Binimetinib Encorafenib

Patient Number	AE CTCAE	AE Grade	AE SAE	AE Intensity	AE Related to
003-004	Neutrophil count decreased	2	No	Unknown	Binimetinib Encorafenib
003-004	Platelet count decreased	1	No	Unknown	Binimetinib Encorafenib
003-005	Fatigue	1	No	Unknown	Binimetinib Encorafenib
003-005	Diarrhoea	1	No	Unknown	Binimetinib Encorafenib
003-005	Diarrhoea	2	No	Unknown	Binimetinib Encorafenib
003-006	Diarrhoea	1	No	Unknown	Binimetinib Encorafenib
003-006	Diarrhoea	1	No	Unknown	Binimetinib Encorafenib
004-003	Vascular disorders - Facial and buccal angioedema	2	No	Unknown	Binimetinib Encorafenib
004-005	Hepatic toxicity	2	No	Unknown	Binimetinib Encorafenib
004-005	Skin and subcutaneous tissue disorders - Rash	2	No	Unknown	Binimetinib Encorafenib
005-003	Retinal tear	2	No	Unknown	Binimetinib
005-003	Fatigue	3	No	Unknown	Encorafenib
006-007	Rash pustular	UK	No	Unknown	Binimetinib Encorafenib
007-001	Alanine aminotransferase increased	3	No	Unknown	Binimetinib Encorafenib
007-001	Aspartate aminotransferase increased	3	No	Unknown	Binimetinib Encorafenib
007-004	Fever	2	No	Unknown	Binimetinib Encorafenib
007-004	Nausea	1	No	Unknown	Binimetinib Encorafenib
007-004	Papulopustular rash	1	No	Unknown	Binimetinib Encorafenib
007-006	Creatinine increased	3	Yes	Significant medical event	Binimetinib Encorafenib
007-008	Uveitis	2	No	Unknown	Binimetinib Encorafenib
009-002	Dry eye	1	No	Unknown	Encorafenib
009-002	Diarrhoea	2	No	Unknown	Binimetinib Encorafenib
010-001	Diarrhoea	2	No	Unknown	Binimetinib Encorafenib
010-001	Fever	2	No	Unknown	Binimetinib Encorafenib
010-001	Vomiting	1	No	Unknown	Binimetinib Encorafenib
010-002	Fever	2	No	Unknown	Binimetinib Encorafenib
010-002	Fever	3	No	Unknown	Binimetinib Encorafenib
010-002	Alopecia	1	No	Unknown	Binimetinib Encorafenib

Patient Number	AE CTCAE	AE Grade	AE SAE	AE Intensity	AE Related to
010-002	Nausea	1	No	Unknown	Binimetinib Encorafenib
010-002	Vomiting	1	No	Unknown	Binimetinib Encorafenib
010-002	Nausea	1	No	Unknown	Binimetinib Encorafenib
010-002	Fever	2	No	Unknown	Binimetinib Encorafenib
010-002	Alanine aminotransferase increased	3	No	Unknown	Binimetinib Encorafenib
010-004	Retinopathy	2	No	Unknown	Binimetinib Encorafenib
010-004	Retinopathy	2	No	Unknown	Binimetinib Encorafenib
010-004	Fatigue	1	No	Unknown	Binimetinib Encorafenib
010-006	Diarrhoea	1	No	Unknown	Encorafenib
010-006	Skin and subcutaneous tissue disorders - Intermittent erythematous lesions on extremities	1	No	Unknown	Binimetinib Encorafenib
010-006	Fatigue	1	No	Unknown	Binimetinib Encorafenib
010-006	Constipation	1	No	Unknown	Binimetinib Encorafenib
010-007	Diarrhoea	1	No	Unknown	Binimetinib Encorafenib
010-007	Nausea	1	No	Unknown	Binimetinib Encorafenib
010-010	Diarrhoea	3	No	Unknown	Binimetinib Encorafenib
010-011	Diarrhoea	3	No	Unknown	Binimetinib Encorafenib
010-015	Alanine aminotransferase increased	2	No	Unknown	Binimetinib Encorafenib
010-015	Aspartate aminotransferase increased	3	No	Unknown	Binimetinib Encorafenib
010-015	Alanine aminotransferase increased	3	No	Unknown	Binimetinib Encorafenib
010-015	Aspartate aminotransferase increased	3	No	Unknown	Binimetinib Encorafenib
010-015	Skin and subcutaneous tissue disorders - Cutaneous toxicity	2	No	Unknown	Binimetinib Encorafenib
011-004	Cecal haemorrhage	4	Yes	Hospitalisation	Binimetinib Encorafenib
013-001	Diarrhoea	1	No	Unknown	Binimetinib Encorafenib
013-001	Hypertension	3	No	Unknown	Binimetinib
013-002	Fatigue	3	No	Unknown	Binimetinib Encorafenib
013-004	Alanine aminotransferase increased	2	No	Unknown	Binimetinib Encorafenib
013-005	Alanine aminotransferase increased	2	No	Unknown	Binimetinib Encorafenib

Patient Number	AE CTCAE	AE Grade	AE SAE	AE Intensity	AE Related to
014-001	Diarrhoea	2	No	Unknown	Binimetinib Encorafenib
014-001	Rash acneiform	1	No	Unknown	Binimetinib Encorafenib
014-001	Dry skin	1	No	Unknown	Binimetinib Encorafenib
014-003	Diarrhoea	2	No	Unknown	Binimetinib Encorafenib
015-001	Alanine aminotransferase increased	3	No	Unknown	Binimetinib Encorafenib
015-001	Aspartate aminotransferase increased	3	No	Unknown	Binimetinib Encorafenib
016-001	Fatigue	2	No	Unknown	Encorafenib
016-003	Diarrhoea	3	Yes	Hospitalisation	Binimetinib Encorafenib
016-003	Hyponatremia	3	Yes	Hospitalisation	Binimetinib Encorafenib
016-006	Creatinine increased	2	No	Unknown	Binimetinib Encorafenib
016-006	Chronic kidney disease	2	No	Unknown	Binimetinib Encorafenib
016-007	Fatigue	2	No	Unknown	Encorafenib
016-007	Diarrhoea	2	No	Unknown	Encorafenib
017-001	Skin and subcutaneous tissue disorders - Vitiligo	2	No	Unknown	Encorafenib
018-001	Fatigue	2	No	Unknown	Binimetinib Encorafenib
018-001	Dyspepsia	2	No	Unknown	Binimetinib Encorafenib
018-003	Fatigue	1	No	Unknown	Binimetinib Encorafenib
018-003	Alopecia	1	No	Unknown	Binimetinib Encorafenib
019-004	Alanine aminotransferase increased	3	No	Unknown	Binimetinib Encorafenib
019-004	Aspartate aminotransferase increased	3	No	Unknown	Binimetinib Encorafenib
019-005	Fatigue	1	No	Unknown	Binimetinib Encorafenib
019-006	GGT increased	3	No	Unknown	Binimetinib Encorafenib
019-006	Fatigue	1	No	Unknown	Binimetinib Encorafenib
019-007	Diarrhoea	2	No	Unknown	Encorafenib
019-007	Abdominal pain	2	No	Unknown	Encorafenib
020-001	CPK increased	1	No	Unknown	Encorafenib
021-004	Hepatobiliary disorders - Hipertransaminasemia	3	Yes	Significant medical event	Binimetinib Encorafenib
